# *Weizmannia coagulans* BC99: A Novel Adjunct to Protein Supplementation for Enhancing Exercise Endurance and Reducing Fatigue

**DOI:** 10.3390/foods14050801

**Published:** 2025-02-26

**Authors:** Minghan Guo, Lina Zhao, Li Cao, Xuan Li, Jie Zhang, Yao Dong, Ying Wu, Shaobin Gu

**Affiliations:** 1 College of Food and Bioengineering, Henan University of Science and Technology, Luoyang 471000, China; guomh321@163.com (M.G.); zhaolina@haust.edu.cn (L.Z.); xbcaoli@163.com (L.C.); lixuan1208@gmail.com (X.L.); zhangjie182@haust.edu.cn (J.Z.); 2 National Demonstration Center for Experimental Food Processing and Safety Education, Luoyang 471023, China; 3 Germline Stem Cells and Microenvironment Lab, College of Animal Science and Technology, Nanjing Agricultural University, Nanjing 210095, China; 4 Henan Engineering Research Center of Food Microbiology, Luoyang 471000, China

**Keywords:** probiotic, protein, anti-fatigue, short-chain fatty acids, *Weizmannia coagulans* BC99

## Abstract

Adequate protein consumption is essential for optimal physical fitness and enhancing athletic performance. This study explored the impact of *Weizmannia coagulans* BC99 on protein-supplemented male fatigued mice, examining aspects such as protein digestion, exercise endurance, fatigue-related biochemistry, oxidative stress, and gut microbiota alterations. Results indicate that the synergistic effect of probiotics and protein significantly boosts the activity of protein-digesting enzymes, enhances protein absorption, and reduces serum levels of urea nitrogen, lactate, lactate dehydrogenase, creatine kinase, malondialdehyde, and the inflammatory cytokines interleukin-1β and interleukin-6 in skeletal muscle. Additionally, serum catalase, glutathione, superoxide dismutase levels, interleukin-4 in skeletal muscle, and glycogen stores in muscle and liver were notably increased. The study also found elevated mRNA expression levels of *Nrf2* and *HO-1* in skeletal muscle. Furthermore, an increase in short-chain fatty acids was observed in the probiotic treatment group, and 16S rDNA sequencing revealed that *Weizmannia coagulans* BC99 enhanced gut microbiota diversity and augmented beneficial bacterial populations including *Roseburia*, *Mucispirillum*, *Rikenella*, and *Kineothrix*. Collectively, these findings suggest that combining BC99 with protein supplementation can effectively improve gut flora, thereby enhancing exercise capacity and exerting potent anti-fatigue effects. Our research provides a new possibility for alleviating exercise-induced fatigue.

## 1. Introduction

Exercise-induced fatigue is a non-pathological state triggered by excessive physical exertion, resulting in diminished muscle performance [1]. This type of fatigue is primarily driven by the depletion of energy substrates, accumulation of metabolic byproducts, and oxidative stress [2,3]. Intensive and prolonged physical activities place substantial stress on various organs, tissues, and cells, thereby reducing work efficiency and athletic performance [4]. Such a decline can profoundly affect the daily functioning of individuals in physically demanding professions including pilots, soldiers, firefighters, and athletes.

In recent years, sports nutrition supplementation has become essential for enhancing exercise capabilities and alleviating fatigue, playing a crucial role in evidence-based training regimens [5]. Among these supplements, proteins are particularly favored by those experiencing exercise fatigue. Proteins, vital for numerous metabolic functions such as hormone regulation and muscle synthesis, become increasingly important under conditions of high-intensity and prolonged exercise to sustain adequate energy production [6]. The type and source of protein supplements significantly influence their effectiveness in improving exercise capacity and muscle mass [7,8]. In the digestive process, proteins that are not broken down in the small intestine are metabolized by gut microbiota in the large intestine into various metabolites, including free amino acids. These amino acids are then absorbed by the gut microbiota to maintain a symbiotic balance with the host, thus, playing a beneficial role [9]. Accordingly, protein supplements enriched with probiotics may offer enhanced health benefits by facilitating protein hydrolysis and amino acid absorption [10].

*W. coagulans* BC99, a spore-forming probiotic, has garnered significant attention in recent research for its robust tolerance to harsh environments and notable probiotic characteristics [11]. Unlike traditional probiotics such as lactobacilli and bifidobacteria, which struggle to survive in extreme conditions and often have low survival rates through the gastric system [12], spore-forming bacteria like *Weizmannia coagulans* exhibit substantial resilience against adverse environments [13]. This bacillus can traverse the digestive tract with relative ease and subsequently germinate and proliferate in the intestine. Recent research highlights *Weizmannia coagulans’* diverse biological actions, including enhancing intestinal digestion, exerting antibacterial effects, regulating the gut microbiota, and conferring benefits to the host’s immune system [14]. However, the specific impact of *W. coagulans* BC99 on physical performance, particularly its potential anti-fatigue properties, has yet to be explored. This study introduces three different concentrations of this probiotic strain to mice: 10^6^ CFU/g·bw/day, 10^7^ CFU/g·bw/day, and 10^8^ CFU/g·bw/day, aiming to assess *W. coagulans* BC99’s efficacy in enhancing protein digestion, improving exercise performance, and mitigating fatigue. The study also seeks to analyze changes in the gut microbiota to elucidate the underlying mechanisms.

## 2. Materials and Methods

### 2.1. Chemicals and Materials

The *Weizmannia coagulans* BC99 strain and protein powder were provided by Wecare Probiotics Co., Ltd. (Suzhou, China). Pepsin (A080-1-1), trypsin (A080-2), lactic acid (LD, A019-2-1), creatine kinase (CK, A032-1-1), urea nitrogen (BUN, C013-2-1), malondialdehyde (MDA, A003-1-1), lactic dehydrogenase (LDH, A020-2), superoxide dismutase (SOD, A001-1-1), glutathione (GSH, A006-1), catalase (CAT, A007-1-1), and glycogen (A043-1-1) were purchased from Nanjing Jiancheng Technology Co., Ltd. (Nanjing, China). Interleukin-4 (IL-4, BYGR507984), Interleukin-6 (IL-6, BYHS101006), and Inter-leukin-1β (IL-1β BYHS500007) were purchased from Nanjing Byabscience Co., Ltd. (Naning, China). SteadyPure RNA Extraction Kit (AG21017-S), Evo M-MLV RT Premix for qPCR (AG11706), and SYBR Green Premix Pro Taq HS qPCR Kit (AG11701) were purchased from Accurate Biology Engineering Co., Ltd. (Changsha, China). Primer design was handled by Bioengineering (Shanghai, China) Co., Ltd.

### 2.2. Animals and Treatment

**Figure 1:**
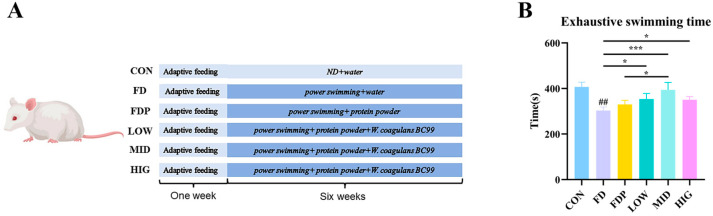
(**A**) The experimental design of this study. (**B**) Impact of BC99 on swimming endurance. Variations were analyzed using ANOVA with the following notations: * *p* < 0.05 signs BC99 groups versus FD or FDP group; ## *p* < 0.01 signs CON group vs. FD group; *** *p* < 0.001 signs BC99 groups versus FD or FDP group.

Seventy-two male Kunming (KM) mice, weighing 20 ± 2 g and aged six weeks, were obtained from Si Pei Fu (Beijing) Biotechnology Co., Ltd. (Beijing, China). The mice were housed in a specific pathogen-free environment with a 12 h alternating light–dark cycle at a temperature range of 22–24 °C. They had unrestricted access to food and water. After the end of the adaptation period (seven days) the seventy-two mice were stochastically separated into six groups (*n* = 12), comprising the blank control group (CON), the fatigue model group (FD), the low-dose BC99 group (LOW, 10^6^ CFU/g·bw/day), the medium-dose BC99 group (MID, 10^7^ CFU/g·bw/day), the high-dose BC99 group (HIG, 10^8^ CFU/g·bw/day), and the positive control group (FDP, protein powder, 2.5 mg/g·bw/day). The BC99 group received the same dose of protein powder by gavage. The CON group received an equal volume of saline by gavage, while BC99 and protein powder were dissolved in saline for administration. All mice received the respective solutions by gavage once a day, except for the basal diet. Gavage was performed at the same time each day, specifically at 8 am. All groups of mice, except the normal control group, underwent weighted swimming exercise for 120 min per day. After six weeks, each mouse participated in rigorous, exhaustive swimming trials. The experimental procedure is shown in Figure 1A.

### 2.3. Exhaustion Swimming Experiment

Following 6 weeks of treatment, six groups of mice (*n* = 12) participated in an exhaustive swimming test. Each mouse was fitted with a lead weight attached to the base of the tail, equivalent to 5% of its body weight, as specified in prior studies [15]. The test was conducted in a 25 cm × 25 cm × 30 cm swimming pool maintained at a temperature of 25 °C. The mice were placed individually into the pool without any support, and the water was constantly agitated to promote sustained swimming activity. A mouse was deemed exhausted if it submerged its nasal tip in water for more than 8 s, at which point the total swimming time until exhaustion was recorded. Immediately following the test, the mice were carefully removed from the water, gently dried with a towel, and returned to their cages for recovery.

### 2.4. Biochemical Analysis

After 6 weeks of treatment, mice were anesthetized using 4% chloral hydrate. Following anesthesia, gentle pressure was applied to the periorbital region to induce proptosis and congestion of the eyeballs, facilitating blood collection. Concurrently, the heart rate was increased by gentle compression using the middle finger of the left hand to expedite blood pumping. Blood collection ceased prior to euthanization via cervical dislocation. Fresh blood samples were collected into heparin sodium anticoagulant tubes and promptly centrifuged (at 4 °C, 3500 rpm, for 20 min). The supernatant was harvested and stored at −80 °C for later analysis of blood biochemical markers, including lactate dehydrogenase (LDH), lactate (LA), creatine kinase (CK), blood urea nitrogen (BUN), superoxide dismutase (SOD), malondialdehyde (MDA), glutathione (GSH), and catalase (CAT) [16,17]. The cecal portion of the small intestine was excised and similarly stored at −80 °C for the assessment of digestive enzyme activities, specifically pepsin and trypsin. Following euthanasia, the soleus muscle was rapidly excised, flash-frozen in liquid nitrogen, and preserved at −80 °C. Glycogen levels in both liver and soleus muscle tissues were quantified according to standard kit protocols. Levels of interleukins IL-4, IL-6, and IL-1β in skeletal muscle were measured using commercially available kits, following the manufacturer’s instructions. Fresh fecal samples were collected from each mouse an hour before dissection and stored at −80 °C for analysis of short-chain fatty acid content [18]. After dissection, cecal contents from each group were collected and stored at −80 °C in centrifuge tubes for subsequent intestinal microbiota sequencing analysis.

### 2.5. Determination of the Expression Levels of Antioxidant Genes Nrf2 and HO-1 in Skeletal Muscle

According to the producer’s instructions, total RNA was extracted from muscle tissue using the SteadyPure RNA Extraction Kit (Accurate Biology Engineering Co., Ltd. Changsha, China), cDNA was amplified in a total volume of 10 μL reaction mixture containing 500 ng template total RNA, 2 μL 5 × Evo M-MLVRT Master Mix and RNase-free water to adjust the volume. The sequences of the primers used in the assays are shown in Table 1. RT-PCR was performed in a total reaction volume of 20 μL, which comprised 100 ng of template cDNA, 10 μL of 2 × SYBR Green Pro Taq HS Premix, 0.4 μL of each primer, and RNase-free water for volume adjustment. The qPCR SYBR Green Pro Taq HS Premix was used for real-time PCR under the following conditions: 95 °C for 30 s followed by 40 cycles at 95 °C for 5 s and 60 °C for 30 s. The expression β-actin was used as a loading control. Target genes were expressed as fold change relative to the control group using the 2^−ΔΔCt^ technique.

### 2.6. Determination of Short-Chain Fatty Acids

The 0.20 g of fresh feces was thoroughly mixed with 1.6 mL of sterile deionized water and allowed to stand at room temperature for 20 min. It was then centrifuged at 15,000 rpm for 15 min at 4 °C. After centrifugation, the supernatant was collected, and the process was repeated. The supernatants from both rounds were combined, filtered through a 0.22 μm membrane filter, and stored at 4 °C for subsequent analysis. For gas chromatography (GC) analysis, 0.20 mL of the supernatant was mixed with 0.70 mL of sterile water, followed by the addition of 0.10 mL of *n*-butanol at a concentration of 100 g/mL. The analysis was conducted using a Thermo TSQ 9000 (Thermo Fisher Scientific, Singapore)with a JN-5MS column (30 m × 0.25 mm, 0.25 µm) to determine the short-chain fatty acids (SCFAs) in mouse feces [19]. The GC conditions were set as follows: the injector temperature was maintained at 250 °C. The initial column temperature was held at 40 °C for 1 min, then increased to 60 °C at a rate of 8 °C/min for another minute, followed by a rise to 70 °C at 10 °C/min for 1 min, and finally escalated to 220 °C at 20 °C/min, where it was held for 10 min. The injection volume was 1 µL, and the flow rate of the column was set at 1.5 mL/min.

### 2.7. Intestinal Microbiota Analysis

As described by Zhang et al., DNA extraction from various samples was performed using a CTAB-based method [20]. Initially, 1000 μL of CTAB lysis buffer was transferred into a 2.0 mL Eppendorf tube, followed by the addition of 20 μL of lysozyme and an appropriate amount of the sample. The mixture was incubated in a water bath at 65 °C for 2–3 h to ensure complete lysis. After incubation, the mixture was centrifuged to collect the supernatant, which was then treated with an equal volume of phenol, chloroform, and isoamyl alcohol (25:24:1). The mixture was centrifuged again after thorough mixing, and the resulting supernatant was further extracted with a chloroform and isoamyl alcohol mixture (24:1). Following another centrifugation, the supernatant was transferred to a 1.5 mL centrifuge tube. To precipitate DNA, three-quarters of the supernatant’s volume of isopropanol was added, mixed thoroughly, and stored at −20 °C. Post-precipitation, the sample was centrifuged, the supernatant was discarded, and the pellet was washed twice with 1 mL of 75% ethanol. After the final centrifugation, the remaining liquid was removed and the DNA was re-suspended in 51 μL of ddH_2_O. RNA contamination was removed by adding 1 μL of RNase A and incubating at 37 °C for 15 min. PCR amplification was carried out in a 25 μL reaction mixture containing 25 ng of template DNA, 12.5 μL of PCR premix, and 2.5 μL of both upstream and downstream primers, with the volume adjusted to 25 μL with PCR-grade water. PCR conditions were set as follows: an initial denaturation at 98 °C for 30 s, followed by 32 cycles of 98 °C for 10 s, annealing at 54 °C for 30 s, and extension at 72 °C for 45 s, with a final extension at 72 °C for 10 min. PCR products were purified using AM Pure XT (Beckman Coulter Genomics, Danvers, MA, USA) beads and quantified with a Qubit fluorometer (Invitrogen, Waltham, MA, USA). Subsequently, libraries were sequenced on a NovaSeq PE250 platform. Alpha and beta diversity metrics were computed using QIIME2, and graphical representations were produced using the R package (v3.5.2). Sequence alignment was performed using BLAST (Lastal 1.0), and feature sequences were annotated with the SILVA database. Additional visualizations were generated using the same version of the R package.

### 2.8. Data Analysis

GraphPad Prism 8.0 was utilized for graphical representations. Statistical analyses were performed using SPSS 19.0. The experimental data are presented as mean ± standard deviation and 95% confidence intervals (*n* = 5). For multi-group comparisons: If the data meet the assumptions of homogeneity of variance and normal distribution, a one-way analysis of variance (ANOVA) is conducted. In cases where the data do not satisfy these criteria, the Kruskal–Wallis test is applied. A *p*-value of <0.05 indicates a statistically significant difference, <0.01 denotes a highly significant difference, and <0.001 reflects an extremely significant difference.

## 3. Results

### 3.1. Effect of BC99 on Swimming Exhaustion Time in Exercise-Fatigued Mice

As depicted in Figure 1B, the FD group exhibited significantly shorter swimming durations compared to the CON group (*p* < 0.01). Following the BC99 intervention, both the LOW and HIG groups demonstrated considerably extended swimming times compared to the FD group, with the BC99 group’s overall showing a notable increase in endurance (*p* < 0.05). Specifically, the swimming time for the MID group reached 394 s, which was approximately 1.3 times longer than that of the FD group (*p* < 0.001). Moreover, when compared to the FDP group, the MID group exhibited a significant improvement in swimming time (*p* < 0.05).

### 3.2. Effect of BC99 on Protein Digesting Enzyme Activity

**Figure 2:**
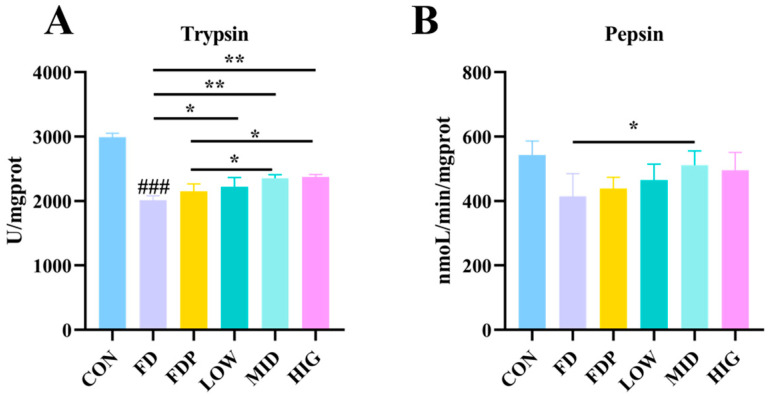
Effect of BC99 on protein-digesting enzyme. (**A**) Trypsin. (**B**) Pepsin. Variations were analyzed using ANOVA with the following notations: * *p* < 0.05 signs BC99 groups versus FD or FDP group; ** *p* < 0.01 signs BC99 groups versus FD or FDP group; ### *p* < 0.001 signs CON group vs. FD group.

As illustrated in Figure 2A,B, trypsin activity significantly decreased in the FD group compared to the CON group (*p* < 0.001). In contrast, trypsin levels were considerably increased in the BC99 group (*p* < 0.05). Additionally, pepsin activity showed a significant enhancement in the MID group (*p* < 0.05). Furthermore, trypsin activity in both the LOW and MID groups was markedly higher compared to the FDP group (*p* < 0.05).

### 3.3. Effects of BC99 on Biochemical Indices of Fatigue in Exercise-Fatigued Mice

**Figure 3:**
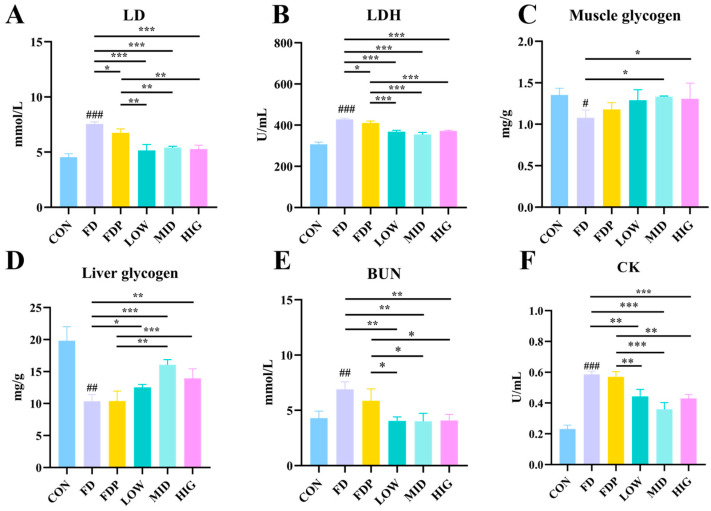
Effect of BC99 on biochemical indices of fatigue in exercise fatigued mice. (**A**) Plasma LD level. (**B**) Plasma LDH level. (**C**) Muscle glycogen. (**D**) Liver glycogen. (**E**) Plasma BUN level. (**F**) Plasma CK level. Variations were analyzed using ANOVA with the following notations: # *p* < 0.05 signs CON group vs. FD group; * *p* < 0.05 signs BC99 groups versus FD or FDP group; ## *p* < 0.01 signs CON group vs. FD group; ** *p* < 0.01 signs BC99 groups versus FD or FDP group; ### *p* < 0.001 signs CON group vs. FD group; and *** *p* < 0.001 signs BC99 groups versus FD or FDP group.

Figure 3A,B demonstrate that the levels of LD and LDH were significantly elevated in the FD group (*p* < 0.001) compared to the CON group. These levels were significantly reduced in the FDP group (*p* < 0.05) and further decreased in the BC99 group (*p* < 0.001) compared to the FD group. Moreover, a notable reduction in both LD and LDH levels was observed in the BC99 group (*p* < 0.01). Figure 3C,D reveal that the concentrations of LG and MG were significantly lower in the FD group (*p* < 0.05) compared to the CON group. Conversely, LG and MG concentrations were significantly increased in the BC99 group (*p* < 0.05) relative to the FD group. Specifically, MG content was substantially elevated in the MID and HIG groups (*p* < 0.001), and LG content was significantly higher in the MID group (*p* < 0.001) compared to the FD group. Additionally, LG values were significantly higher in the MID (*p* < 0.01) and HIG (*p* < 0.001) groups compared to the FDP group. Finally, Figure 3E,F indicate that BUN and CK levels were significantly higher in the FD group (*p* < 0.01) compared to the CON group. These levels were notably decreased in the BC99 groups (*p* < 0.01) compared to the FD group. Both CK (*p* < 0.01) and BUN (*p* < 0.05) levels were significantly reduced in the BC99 group compared to the FDP group, indicating an alleviation of exercise-induced biochemical stress markers.

### 3.4. Effects of BC99 on Oxidative Stress Indices in Exercise-Fatigued Mice

**Figure 4:**
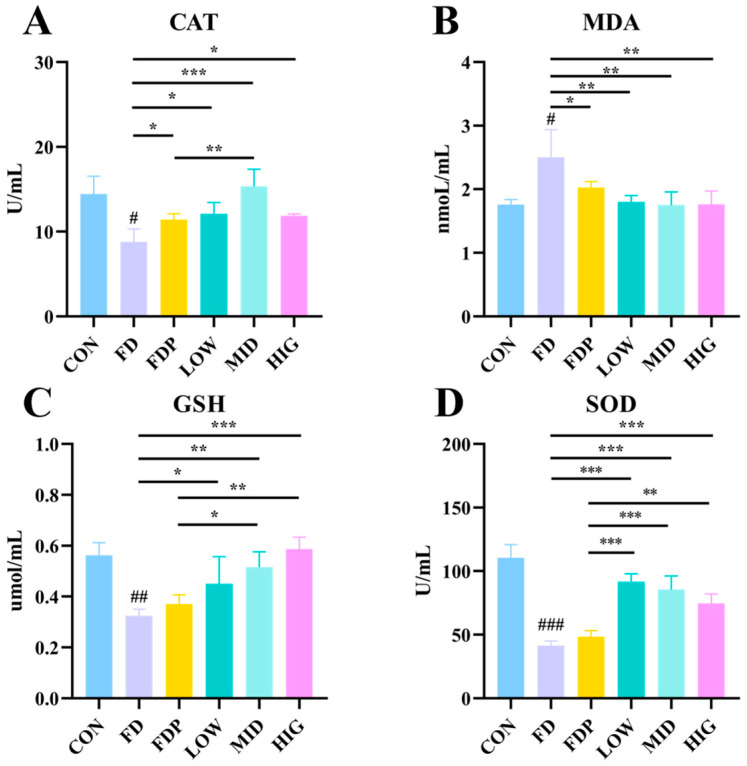
Effect of BC99 on oxidative stress in exercise fatigued mice. (**A**) Plasma CAT level. (**B**) Plasma MDA level. (**C**) Plasma GSH level. (**D**) Plasma SOD level. Variations were analyzed using ANOVA with the following notations: # *p* < 0.05 signs CON group vs. FD group; * *p* < 0.05 signs BC99 groups versus FD or FDP group; ## *p* < 0.01 signs CON group vs. FD group; ** *p* < 0.01 signs BC99 groups versus FD or FDP group; ### *p* < 0.001 signs CON group vs. FD group; and *** *p* < 0.001 signs BC99 groups versus FD or FDP group.

As illustrated in Figure 4A–D, there was a notable reduction in the serum levels of antioxidant enzymes SOD, CAT, and GSH in the FD group (*p* < 0.05) compared to the CON group. Conversely, the content of MDA, an indicator of oxidative stress, exhibited a significant increase in the FD group (*p* < 0.05) relative to the CON group. Both the FDP and BC99 groups showed a significant enhancement in CAT, GSH, and SOD activities, along with a significant reduction in MDA levels when compared to the FD group. Notably, the BC99 group demonstrated significantly higher SOD levels (*p* < 0.01) compared to the FDP group. Furthermore, the MID and HIG groups exhibited significantly higher GSH levels (*p* < 0.01), and the MID group also displayed significantly elevated CAT levels (*p* < 0.01) in comparison to the FDP group. These findings suggest that BC99 supplementation effectively mitigates oxidative stress and enhances antioxidant defense mechanisms in exercise-fatigued mice.

### 3.5. Effects of BC99 on Inflammatory Factors in Skeletal Muscle of Fatigued Mice

**Figure 5:**
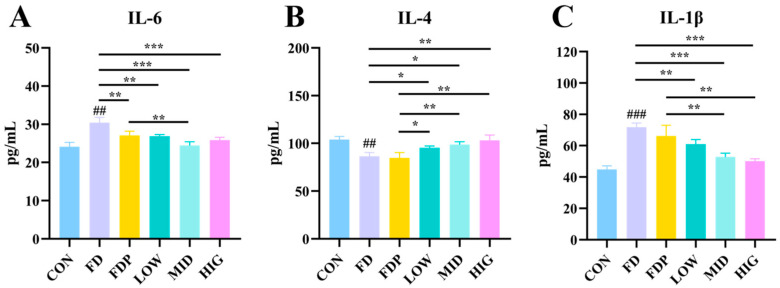
Effect of BC99 on inflammatory factors in exercise fatigued mice. (**A**) Muscle IL-6 level. (**B**) Muscle IL-4 level. (**C**) Muscle IL-1β level. Variations were analyzed using ANOVA with the following notations: * *p* < 0.05 signs BC99 groups versus FD or FDP group; ## *p* < 0.01 signs CON group vs. FD group; ** *p* < 0.01 signs BC99 groups versus FD or FDP group; ### *p* < 0.001 signs CON group vs. FD group; and *** *p* < 0.001 signs BC99 groups versus FD or FDP group.

The levels of muscles IL-6 and IL-1β were markedly elevated (*p* < 0.01), while IL-4 levels were considerably reduced (*p* < 0.01) in the FD group of mice compared to the CON group, as depicted in Figure 5A–C. These findings imply that excessive exercise induces heightened inflammatory responses in mice. In comparing the MID and HIG groups with the FD group, it was evident that the muscle levels of IL-6 and IL-1β were considerably lower (*p* < 0.001) in the MID and HIG groups. Additionally, the levels of IL-4 were found to be significantly higher (*p* < 0.05) in the LOW and MID groups, and they were notably higher (*p* < 0.01) in the HIG group. Furthermore, in comparison to the FDP group, the MID group exhibited considerably lower levels of IL-1β and IL-6 (*p* < 0.01), while the BC99 group showed considerably higher levels of IL-4 (*p* < 0.05).

### 3.6. Effect of BC99 on mRNA Expression Levels of Genes Related to Nrf2 Signaling Pathway in Skeletal Muscle

**Figure 6:**
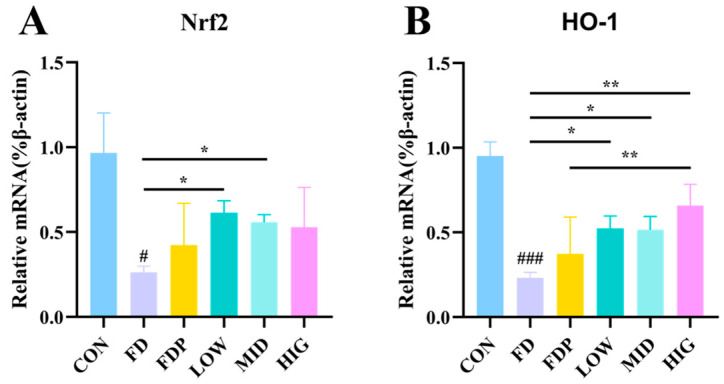
Effect of BC99 on mRNA expression levels of genes related to *Nrf2* signaling pathway in skeletal muscle. (**A**) *Nrf2*. (**B**) *HO-1*. Variations were analyzed using ANOVA with the following notations: # *p* < 0.05 signs CON group vs. FD group; * *p* < 0.05 signs BC99 groups versus FD or FDP group; ** *p* < 0.01 signs BC99 groups versus FD or FDP group; and ### *p* < 0.001 signs CON group vs. FD group.

As shown in Figure 6A,B, the mRNA levels of *Nrf2* and *HO-1* in the skeletal muscles of mice in the FD group were notably lower than the CON group (*p* < 0.05). In contrast, the mRNA expression levels of *Nrf2* and *HO-1* in the muscles of mice in the BC99 group were significantly higher than those in the FD group (*p* < 0.05). This suggests that the addition of BC99 activated the *Nrf2* pathway in the muscles of mice, leading to an increased mRNA expression of the antioxidant enzyme *HO-1*. Additionally, the levels of *HO-1* were significantly elevated in the HIG group compared to the FDP group (*p* < 0.01).

### 3.7. Improvement of BC99 on SCFAs in Mice Intestine

**Figure 7:**
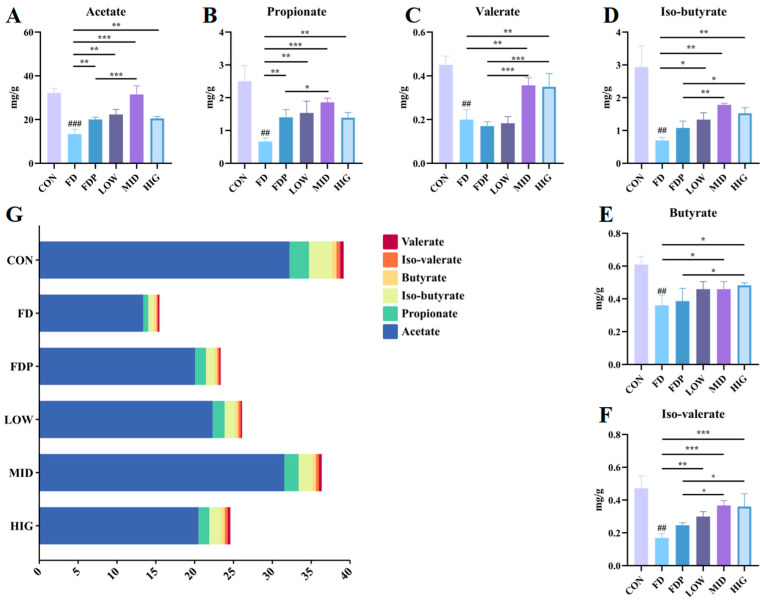
Effect of BC99 on changes in SCFAs in the feces of fatigued mice. (**A**) Acetate, (**B**) Propionate, (**C**) Valerate, (**D**) Iso-butyrate, (**E**) Butyrate, (**F**) Iso-valerate, (**G**) Total SCFAs content. Variations were analyzed using ANOVA with the following notations: * *p* < 0.05 signs BC99 groups versus FD or FDP group; ## *p* < 0.01 signs CON group vs. FD group; ** *p* < 0.01 signs BC99 groups versus FD or FDP group; ### *p* < 0.001 signs CON group vs. FD group; and *** *p* < 0.001 signs BC99 groups versus FD or FDP group.

As shown in Figure 7A–F, the content of SCFA in the feces of mice in the FD group (*p* < 0.01) was considerably reduced compared to the CON group. The concentrations of acetate, propionate, iso-butyrate and iso-valerate showed a notably higher level in the BC99 group when compared to the FD group (*p* < 0.05), while the levels of butyrate (*p* < 0.05) and valerate (*p* < 0.01) were significantly increased in the MID and HIG groups. In comparison with the FDP group, the MID group exhibited a substantially elevated level of acetate (*p* < 0.001), propionate (*p* < 0.05), iso-butyrate (*p* < 0.01), iso-valerate (*p* < 0.05), and valerate (*p* < 0.001). Valerate (*p* < 0.001), iso-butyrate (*p* < 0.05), butyrate (*p* < 0.05) and iso-valerate (*p* < 0.05) were considerably increased in the HIG group. When analyzed based on the total SCFA content, as shown in Figure 7G, the MID group had the best effect.

### 3.8. Effects of BC99 on the Composition of the Gut Microbiota

**Figure 8:**
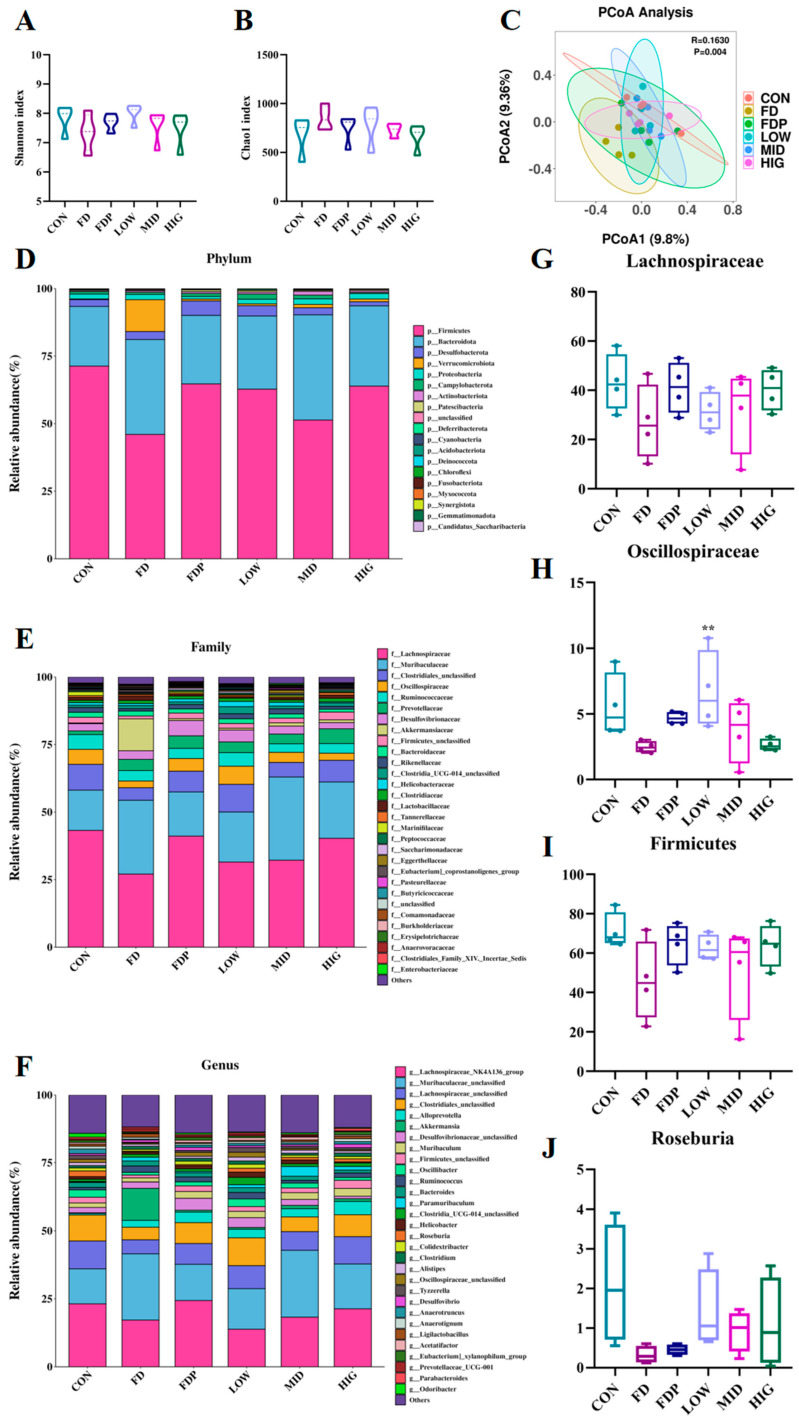
Effects of BC99 on gut microbiota abundance and distribution at the phylum, family and gene levels. (**A**,**B**) α-diversity indices reflected by Chao l and Shannon indices. (**C**) β-diversity of gut microbiota; (**D**–**F**) the relative abundance of gut microbiota at the phylum level for each mouse is demonstrated through the utilization of stacked column bar graphs. (**G**–**J**) Differential strains at the level of different species. Variations were analyzed using ANOVA with the following notations: ** *p* < 0.01 indicates BC99 group vs. FD group.

To assess the influence of BC99 on the gut microbiome of mice, we performed 16S rDNA gene sequencing of the microbiome in the gut contents of the mice. Figure 8A,B illustrates our utilization of the Shannon and Chao1 indices to quantify the species richness of the microbiome and the Shannon and Chao1 indices were not significantly different in the BC99 group compared to the CON group. This indicates that the diversity and species richness of the murine gut microbiota is not statistically significantly different (*p* > 0.05). As shown in Figure 8C, according to beta diversity principal coordinate analysis of the Bray–Curtis distance matrix, the distance between the CON group and FD group mouse samples is relatively far, indicating that fatigue exercise causes intestinal disorders in mice. After 6 weeks of probiotic and protein combined intervention, the BC99 group and FD group mouse samples clustered into relatively different groups. This result suggests that the combined intervention of BC99 and protein can significantly (*p* = 0.004) improve the intestinal microbiota structure of fatigue mice.

Utilizing the sequencing data of the mouse 16S rDNA gene, we conducted an analysis of the variations in bacterial presence within the gut microbiota across different mouse groups at the phylum, family, and genus levels. These variations were visually represented as cumulative histograms showing the relative abundance differences (Figure 8D–J). *Firmicutes*, *Bacteroidetes*, *Desulfobacterota*, and *Verrucomicrobia* accounted for more than 95% of the microbiota composition. The FD group showed a decrease in the mean relative abundance of *Firmicutes* of 25.32% in comparison with the CON group, while the FDP, LOW, and HIG groups showed an increase of 18.72%, 16.77%, and 17.91%, respectively, compared to the FD group. However, there was no statistically significant differences (*p* > 0.05) in the impact of the BC99 group on the abundance of *Firmicutes*. At the family level, the top five groups were *Lachnospiraceae*, *Muribaculaceae*, *Clostridiales_unclassified*, *Oscillospiraceae*, and *Ruminococcaceae*. LOW group relative abundance of *Oscillospiraceae* was considerably greater than that of the FD group (*p* < 0.01). BC99 group relative abundance of *Roseburia* was higher than that of the FD group (*p* > 0.05).

**Figure 9:**
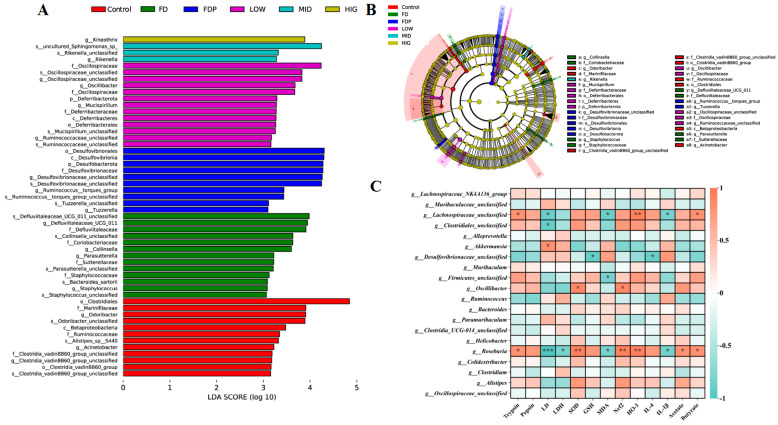
The microbiomes of all groups were characterized through OTU-based LEFSe and LDA analyses. (**A**) Significant differences in bacteria between groups were indicated by LDA scores (log LDA > 3.0; *n* = 4). (**B**) The phylogenetic distribution of colonic microbes associated with each group was shown through Cladograms using the LEFSe method. (**C**) Spearman’s correlation was used for heatmap analysis. * *p* < 0.05, ** *p* < 0.01, *** *p* < 0.001.

To determine the differential strains among all groups, we used linear distance analysis (LDA) based on nonparametric factors KW and rank test to analyze the effect size (LEfSe) of the gut microbiota species. Figure 9 shows species with significant differences, and LDA scores greater than 3.0 indicate the degree of influence of species with significant differences between groups. As shown in Figure 9A, the CON group contained 12 biomarkers such as *o_Clostridiales* and *f_Muribaculaceae*; the FD group contained 13 biomarkers such as g_Defluviitaleaceae_UCG_011 and *g_Parasutterella*; the FDP group contained 10 biomarkers such as *o_Desulfovibrionia*, *c_Desulfovibrionia*, and *p_Desulfvbacterota*; the LOW group included *f_ Oscillospiraceae*, *g_Oscillibacter*, *p_Deferribacterota*, and 13 other biomarkers; the MID group contained three biomarkers including *g_Rikenella*; and the HIG group contained one biomarker, *g_Kineothrix*. The results showed significant alteration in the gut microbiota composition in all groups after administration of different doses of BC99. LEfSe multilevel strain identification showed that compared with the CON group, the FD group had a decrease in the content of beneficial bacteria such as *f_Marinifilaceae*, *g_Odoribacter*, and *f_Ruminococcaceae*, and BC99 intervention produces a wide range of beneficial bacteria such as *Mucispirillum*, *Rikenella*, and *Kineothrix*, with each beneficial strain being dominant in different dose groups.

Spearman’s correlation analysis explored the correlation between gut motility, oxidative stress, inflammation, and SCFAs with gut flora indicators in mice. As shown in Figure 9C, the abundance of *g_Lachnospiraceae_unclassified* is positively associated with trypsin, *HO-1*, and propionate, and it is negatively associated with LD, MDA, and IL-1β; the abundance of *g_Desulfovibrio_unclassified* is negatively associated with GSH and IL-4; the abundance of *g_Firmicutes_unclassified* is negatively associated with MDA; the abundance of *Roseburia* is positively correlated with trypsin, SOD, *Nrf2*, *HO-1*, acetate, and propionate, and it is negatively associated with LD, LDH, MDA, and IL-1β; the abundance of *Oscillibacter* is positively associated with SOD and *Nrf2*.

## 4. Discussion

Exercise fatigue is a condition of physical exhaustion induced by prolonged and intense physical activity or training. Recent studies have shown that such activities can significantly influence the abundance and expression of gut microbiota [21]. In light of this, probiotics have increasingly been incorporated into sports nutrition, particularly among athletes. Their role is to modulate and enhance the gut microbiota composition, potentially offering various physiological advantages that contribute positively to athletic performance and general well-being [10,22]. This study aimed to evaluate the effects of BC99 on protein digestion, as well as the combined impact of BC99 and protein on exercise capacity and anti-fatigue responses in fatigued mice. The results demonstrated that BC99 supplementation markedly improved the activity of protein-digesting enzymes, thereby enhancing protein digestion and absorption in mice. The weight-bearing swimming test, a widely used method to assess the anti-fatigue properties of substances, showed that longer swimming durations are indicative of greater anti-fatigue effectiveness [23]. Compared to sole protein supplementation, the concurrent use of *W. coagulans* BC99 and protein significantly enhanced the exercise endurance and antioxidant capacity of tired mice, while also modifying the composition of gut microbiota. Additionally, the resistance to fatigue is closely linked to alterations in the abundance of crucial gut bacteria and levels of short-chain fatty acids (SCFAs). These findings suggest that integrating probiotics like BC99 with protein supplements could be a strategic approach to enhance athletic performance and mitigate fatigue.

Supplementing protein is crucial for athletes, serving not only to alleviate muscle soreness post-exercise but also to enhance physical recovery and performance across various aspects [24]. Adequate protein intake aids in effectively repairing muscle fibers damaged during exercise, promoting muscle function recovery, and improving subsequent performance [25]. It is noteworthy that increased protein consumption can affect the gut microbiota of athletes, especially under conditions of metabolic stress or heightened metabolic demands. Certain microorganisms, such as *W. coagulans* NJ0516, can indirectly boost protein absorption and utilization by enhancing the intestinal environment [26]. Chang et al. explored the impact of condensed *W. coagulans* NRS609 on the growth performance and intestinal health of carp, revealing that *W. coagulans* NRS609 significantly enhances the digestive enzyme activity in these fish [27]. Beyond merely aiding nutrient digestion and absorption, these probiotics can also reduce inflammation and foster intestinal health [28]. The growth and development of animals are intrinsically reliant on nutrients, and the efficient digestion and absorption of these nutrients necessitate the presence of intestinal digestive enzymes with high levels of activity [29,30]. Pepsin, a specific digestive enzyme, plays a crucial role in the degradation of proteins, thereby promoting their subsequent absorption and utilization within the body. The principal functions attributed to pepsin are as follows: it actively promotes the digestion of proteins, aids in the efficient absorption of nutrients, partakes in the cooperative actions among various digestive enzymes, and contributes to the maintenance of intestinal health. This enzyme is integral to the overall digestive process, ensuring that proteins are appropriately processed and assimilated, which in turn supports the body’s physiological functions and metabolic activities. In this study, supplementation with BC99 was shown to increase the activity of protein-digesting enzymes, thereby improving protein digestion and absorption in mice, which contributed to more pronounced effects in alleviating fatigue.

Probiotic formulations have demonstrated the ability to alter the composition and functional activities of intestinal bacteria, thereby increasing the diversity of the gut microbiota [31,32]. *W. coagulans* GBI-30, in particular, augments the effects of prebiotics by supporting the growth of beneficial bacteria and enhancing the synthesis of SCFAs [33,34]. During high-intensity exercise, energy is primarily generated through the rapid breakdown of glucose, which can lead to decreased blood glucose levels [35]. The subsequent conversion of MG and LG into glucose helps maintain blood glucose equilibrium [36,37]. However, this sustained energy expenditure results in the accumulation of metabolites such as lactic acid, lactate dehydrogenase, creatine kinase, and blood urea nitrogen, contributing to metabolic imbalances and increased fatigue. High-intensity exercise also produces a significant amount of ROS. An overabundance of ROS can escalate the production of MDA, disrupt cellular metabolism and physiological functions, and ultimately lead to fatigue [38]. Enzymes like SOD and GSH play crucial roles in maintaining the body’s oxidative balance by converting harmful peroxides into harmless hydroxides, thereby protecting cells from oxidative damage [39]. During states of exercise-induced fatigue, the body’s metabolic responses adapt by triggering cells to release inflammatory factors, which initiate an inflammation reaction [40]. The regulation of the *Nrf2* pathway has been shown to enhance the activity of the antioxidant enzyme *HO-1*, found within this pathway, which helps neutralize excess ROS and reduce cellular oxidative stress [41,42]. Previous studies have suggested that probiotics can exert anti-fatigue effects by modulating the gut microbiota [43,44,45]. An increased presence of Bacteroides and Firmicutes in the gut microbiota has been associated with improved oxidative and antioxidative balance and enhanced fatigue metabolism [46]. In this study, BC99 was observed to increase the abundance of Firmicutes, although the difference was not statistically significant (*p* = 0.133). Furthermore, *Mucispirillum*, *Rikenella,* and *Kineothrix* were identified as biomarkers for the BC99 group. *Mucispirillum* has been shown to play a crucial role in protecting against colitis caused by non-typhoidal Salmonella typhimurium in mice [47]. The increased abundance of *Rikenella* contributes to SCFA production, which benefits glucose metabolism [48]. The fermentation byproducts of *Kineothrix*, primarily acetate and butyrate, enhance SCFA synthesis and provide health benefits [49,50]. It has also been shown that bacteria such as *g_Odoribacter* and f_Ruminococcaceae can promote an increase in SCFA content in the intestine, beneficial for overall health. However, a reduction in these beneficial bacteria was noted in the FD group, indicating a decrease in beneficial bacterial content in fatigued mice [51,52].

SCFAs are pivotal in regulating pH levels and facilitating the absorption of essential minerals such as calcium, iron, and magnesium. They also play a crucial role in the metabolism of glucose and protein in the liver [53]. Furthermore, SCFAs are instrumental in maintaining normal intestinal structure and function [54] and serve as the primary energy source for colon cells [55]. As depicted in Figure 8C, a positive correlation exists between the abundance of Roseburia and enzymatic activities of trypsin, SOD, *Nrf2*, *HO-1*, and the levels of acetate and butyrate. Conversely, there is a negative correlation with LD, LDH, MDA, and IL-1β. In this study, the abundance of Roseburia in the BC99 group increased (*p* = 0.053), which corresponded with an increase in butyric acid content among SCFAs. Research has indicated that W. coagulans can induce anaerobic and acidic conditions in the intestine, favorable for the proliferation of SCFA-producing bacteria [56]. Notably, *Faecalibacterium* and *Roseburia* are among the predominant producers of butyric acid [57]. Sodium butyrate, a SCFA, not only enhances the proliferation of beneficial bacteria but also inhibits the growth of detrimental bacteria [58]. In conditions such as irritable bowel syndrome, butyrate exerts significant anti-inflammatory effects by activating anti-inflammatory pathways and suppressing pro-inflammatory host responses [59]. SCFAs serve as an energy source for the liver and muscle cells, enhancing prolonged performance by maintaining stable blood glucose levels [60,61]. Butyric acid maintains blood sugar balance and promotes glycogen metabolism, while propionic acid is a precursor for hepatic glucose synthesis [62,63]. Previous studies have shown that probiotic intake can reduce serum levels of pro-inflammatory cytokines and elevate serum levels of anti-inflammatory cytokines [64]. The addition of different doses of sodium butyrate to calf diets resulted in a linear increase in serum GSH activity with increasing addition, and MDA levels were markedly lower than in the not supplemented group [65]. *Oscillibacter* may be associated with lowering cholesterol levels [66], and the presence of *Oscillibacter* showed a positive association with SOD and *Nrf2* in this research, although the specific mechanism remains unclear. The above research results are similar to the results of this experiment, adding six weeks of *W. coagulans* BC99 and protein significantly increased weight-bearing swimming time, reduced serum fatigue indicators (LD, BUN, LDH, and CK) levels, and increased LG and MG levels. Furthermore, the combination of probiotics and proteins has been shown to markedly increase the antioxidant capacity of mice, decrease the inflammatory response in fatigued mice, and safeguard muscle tissue from damage. As a result, *W. coagulans* BC99 may have a positive influence on combatting fatigue by promoting beneficial microorganisms, suppressing harmful microorganisms, stimulating the production of short-chain fatty acids, and maintaining a healthy balance of gut microbiota.

However, this study is not without its limitations. While the analysis of gut microbiota in mice indicated that BC99 could enhance fatigue recovery and improve exercise capacity, the precise molecular mechanisms underlying these effects remain to be fully elucidated and require further experimental validation. Moreover, although BC99 shows potential as a supplemental protein to enhance exercise endurance in mice, the applicability of these findings to human subjects is not direct and necessitates additional investigation in clinical settings.

## 5. Conclusions

The present study underscores the potential of BC99, combined with protein supplementation, to enhance exercise performance and anti-fatigue effects in mice. These encouraging results highlight the probiotic’s impact on gut health and its subsequent influence on physical endurance. Moving forward, further studies are needed to explore the molecular pathways involved and to establish the efficacy and safety of BC99 in human populations. Ultimately, expanding this research into clinical trials will be crucial to determine the probiotic’s practical benefits for athletes and individuals engaged in physical activities. This could pave the way for new interventions in sports nutrition, potentially offering a natural and effective solution to enhance athletic performance and recovery.

## Figures and Tables

**Table 1:** Primer sequences.

Gene	Primer Sequence
*Nrf2*	F: CTTTAGTCAGCGACAGAAGGAC
	R: AGGCATCTTGTTTGGGAATGTG
	F: AGGTACACATCCAAGCCGAGA
*HO-1*	R: CATCACCAGCTTAAAGCCTTCT
*β-actin*	F: CTGTGTTTTGGTCTTACGGTAC
	R: AAAAAGCCTGTCTGTGATTCAC

## Data Availability

The original contributions presented in this study are included in the article. Further inquiries can be directed to the corresponding authors.
